# Gene methylation of *CADM1 and MAL* identified as a biomarker of high grade anal intraepithelial neoplasia

**DOI:** 10.1038/s41598-022-07258-5

**Published:** 2022-03-03

**Authors:** Samuel Phillips, Kahli Cassells, Suzanne M. Garland, Dorothy A. Machalek, Jennifer M. Roberts, David J. Templeton, Fengyi Jin, I. Mary Poynten, Richard J. Hillman, Andrew E. Grulich, Gerald L. Murray, Sepehr N. Tabrizi, Monica Molano, Alyssa M. Cornall, SPANC team

**Affiliations:** 1 Department of Obstetrics and Gynecology, University of Melbourne, Parkville, VIC 3052 Australia; 2 Centre Women’s Infectious Diseases Research, The Royal Women’s Hospital, Parkville, VIC 3052 Australia; 3 Murdoch Children’s Research Institute, Parkville, VIC 3052 Australia; 4 HIV Epidemiology and Prevention Program, The Kirby Institute, University of New South Wales, Kensington, NSW 2052 Australia; 5 Douglass Hanly Moir Pathology, Macquarie Park, NSW 2113 Australia; 6 Department of Sexual Health Medicine, Sydney Local Health District, Camperdown, NSW 2050 Australia; 7 Discipline of Medicine, Central Clinical School, Faculty of Medicine and Health, The University of Sydney, Sydney, NSW 2006 Australia; 8 Dysplasia and Anal Cancer Services, St Vincent’s Hospital, Darlinghurst, NSW 2010 Australia

**Keywords:** Cancer, Microbiology, Molecular biology

## Abstract

Human papillomavirus (HPV) is detected in up to 96% of anal squamous cell cancers, where screening programs needed. However, the best methodology is still undetermined. Host DNA methylation markers *CADM1, MAL* and *miR124* have been identified in cervical disease, but not anal disease. Anal swabs varying by disease grade were assessed for DNA methylation of *CADM1, MAL* and *miR124-2.* Each marker was compared across disease grades, stratified by HPV and HIV status. Receiver operating characteristic curves identified the predictive value of significant gene candidates. *CADM1* methylation was significantly higher in high-grade squamous intraepithelial lesions (HSIL) compared with low-grade (LSIL) (p = 0.005) or normal (p < 0.001) samples with 67.2% correctly identified as HSIL. *MAL* methylation was significantly (p = 0.002) increased in HSIL compared with LSIL in HIV positive participants with 79.8% correctly indicated as HSIL. Gene *miR124-2,* showed no difference between disease grades. Biomarkers with established diagnostic value in cervical disease have limited utility in the prediction of anal disease, with *CADM1* identified as a marker with screening potential in a gay and bisexual men (GBM) population and *MAL* in HIV positive GBM population. New markers specific to the anal mucosa are required to improve triage of high-risk individuals.

## Introduction

Human papillomavirus (HPV) causes approximately 88% to 96% of anal cancers^1,2^. The majority of anal cancers arise from the squamo-columnar junction of the anal canal and are predominantly anal squamous cell carcinomas (ASCC)^3^.

Rates of ASCC in the general population are low, at less than 2 per 100,000 person years, with slightly higher rates in women compared with men^4^. Incidence has been steadily increasing over the past three decades^5^ although, recent data has suggested ASCC in people with HIV have decreased over the past decade (2008–2012), thought to be due to improved HIV treatments^6^. Vaccination against high-risk HPV (HRHPV) genotypes that cause the majority of anal cancers, when administered prior to HPV exposure, are highly effective in reducing the incidence of the precursor lesion, anal high-grade squamous intraepithelial lesions (HSIL)^7^. However, the progress of introduction of gender neutral vaccine programs is slow^8^, as such a reduction in anal cancer rates will not be seen for many decades^7,9^. Among the high-risk groups predicted to benefit from effective screening, gay and bisexual men (GBM) are the most studied as HIV negative GBM are up to 20 times and HIV positive GBM are up to 100 times more likely to develop anal HSIL and/or ASCC than heterosexual men. Other high-risk populations with increased rates of ASCC include women and heterosexual men with HIV infection (12-fold higher), women with previous lower genital tract disease related to HPV, and transplant recipients^5,10–12^.

Current clinical screening algorithms are based on anal cytology assessment, followed by high-resolution anoscopy (HRA) guided biopsy and histology as a diagnostic tool. Performance of anal cytology as a screening tests for anal cancer prevention has been demonstrated to vary depending on the population and screening protocols to define risk of cancer development^13^. A recent meta-analysis evaluated the performance of anal cytology in women and men and identified an overall sensitivity and specificity of atypical squamous cells of undetermined significance or higher positive cytology for the detection of AIN2 or higher at 77.3% and 55.5%, respectively^14^. Furthermore, the use of HRA-guided biopsies are considered imperfect due to anal mucosal folds and coexisting pathology^15^. There is also a lack of treatment options available. Consequently, there are no universally accepted guidelines for anal screening^14^.

HRHPV DNA detection has been adopted for cervical screening in many countries being more objective and sensitive to cytology^16^. The application of HRHPV DNA detection as a primary test for anal screening in high-risk populations may be limited, due to the high prevalence of anal HRHPV and the presence of multiple HPV types, resulting in high sensitivity but poor specificity^14,17–19^.

Cellular biomarkers are indicative of underlying biological changes as a result of modulation of oncogenic gene regulation during carcinogenesis, and may be used as markers of neoplastic transformation of cells^20^. The methylation of CpG sites for cell adhesion molecule 1 (*CADM1*), T-lymphocyte maturation associated protein (*MAL*) and the micro RNA 124-2 (*miR124-2*) are promising biomarkers in HPV-related cervical intraepithelial neoplasia (CIN)^21^ but have had limited evaluations in HPV-related anal disease, with only a single study investigating miR124-2^22^. These three genes have been previously identified as tumour suppressors, mediators of organised epithelial cell growth and potent immune system regulators^23–29^.

This study aimed to examine the performance of previously identified cervical cancer methylation markers (*CADM1*, *MAL*, and *miR124-2*) in anal HSIL, using samples collected as part of the Study of the Prevention of Anal Cancer (SPANC)^5^. This was used to determine how well these methylation markers predict anal HSIL, as a potential triage test alone and in combination with HRHPV positivity and HIV status.

## Results

### Study participants

From the 617 SPANC participants with collected baseline anal cytology samples, 454 were included in this analysis (4 participants without consent, 86 participants were lost to follow-up, 17 samples with unassessable baseline HPV results and 56 samples with insufficient remaining sample). The median age was 49 years (range 35–75 years) and 36% were HIV positive. Of the total, 429 (94.5%) samples had valid host DNA methylation results (Table 1) and 315 (73.4%) tested positive for HRHPV. The highest degree of abnormality diagnosed was HSIL n = 117 (27%), LSIL n = 26 (6%) and normal n = 286 (66.7%) (Table 1).

**Table 1:** Samples included in analysis categorised by composite disease grade.

Highest grade	Anal swabs [(%) of valid samples]			HRHPV positive [(%) of valid samples]		
	Total	HIV−	HIV+	Total	HIV−	HIV+
HSIL	117 (27)	52 (19)	65 (40)	108 (34)	50 (27)	58 (45)
LSIL	26 (6)	15 (6)	11 (7)	18 (6)	11 (6)	7 (6)
Normal	286(67)	200 (75)	86 (53)	189 (60)	126 (67)	63 (49)
Total valid	429	267	162	315	187	128
Failed methylation	25	15	10	20	11	9
Total tested	454	282	172	335	198	137

### Methylation of the gene *CADM1* differentiates between HSIL and LSIL/normal samples

**Figure 1:**
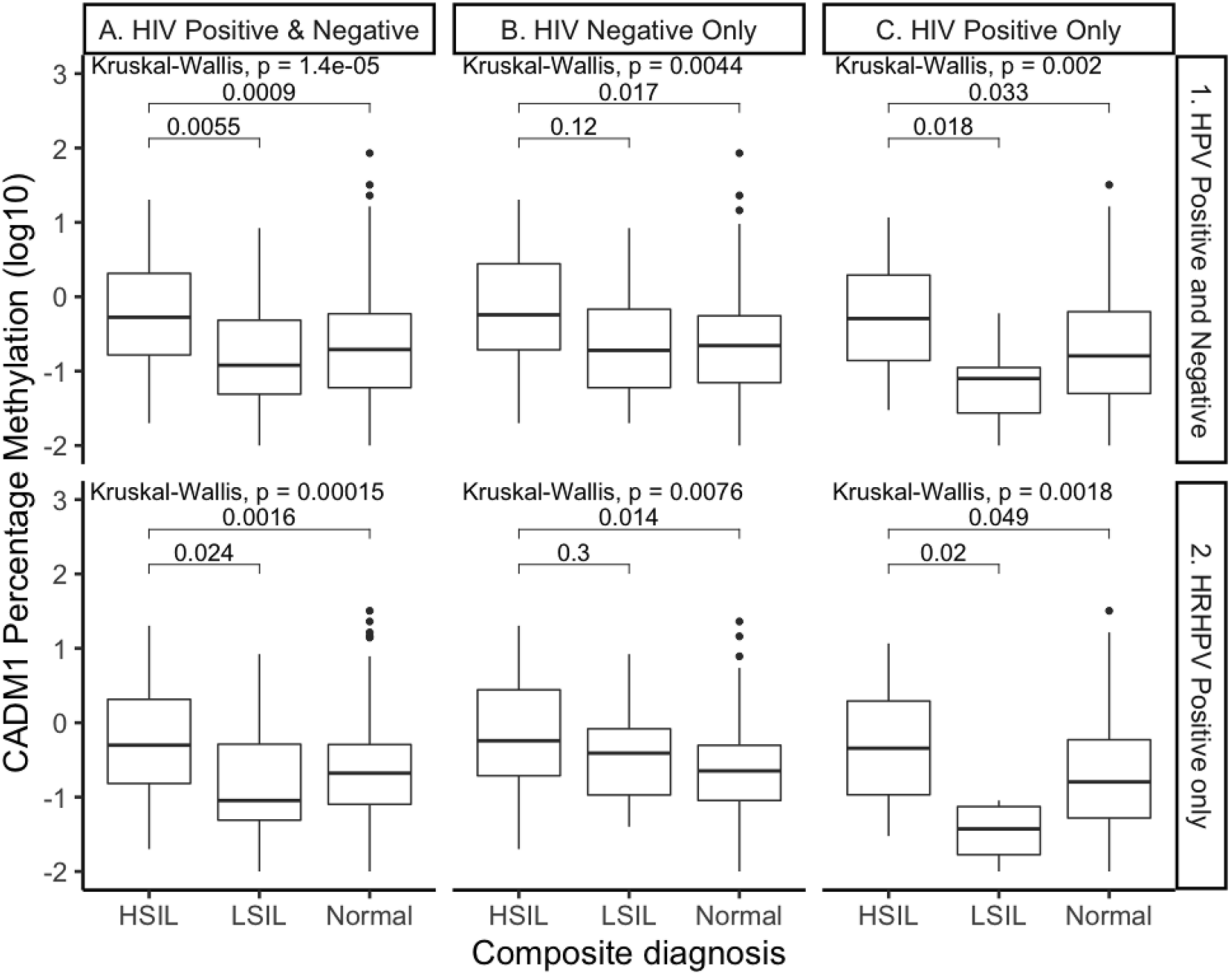
Percentage Methylation of gene *CADM1*. All samples represented by HRHPV positivity and HIV status. Comparisons assessed by the nonparametric Wilcoxon test, with error bars corresponding to the first and third quartiles (the 25th and 75th percentiles).

*CADM1* methylation was significantly different across all samples when stratified by disease (p < 0.001) (Fig. 1, panel A1). A further group specific analysis (Wilcoxon), also revealed that *CADM1* methylation from anal swab samples from men with HSIL was significantly different compared to those with both LSIL (p = 0.005) and normal samples (p < 0.001) (Fig. 1, panel A1). There was also a significant difference between HSIL and LSIL (p = 0.024) and HSIL and normal (p = 0.002) when the analysis was limited to HRHPV positive samples only (Fig. 1, panel A2).

**Figure 2:**
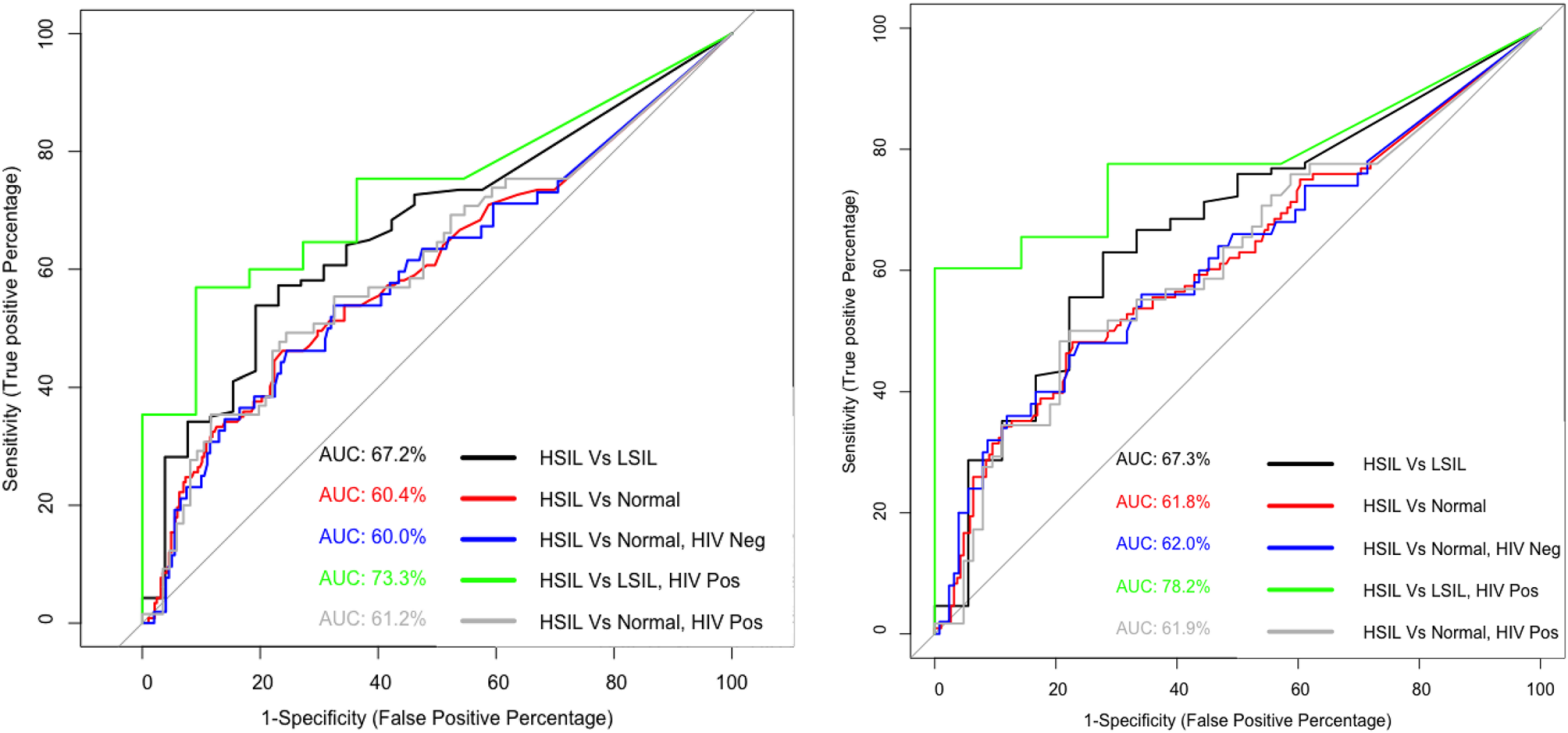
Receiver Operating Characteristic (ROC) curve for the percentage methylation of gene *CADM1* for all significant (p, ≤ 0.05) comparison combinations identified in the nonparametric Wilcoxon test. Comparisons include HSIL versus LSIL (black), HSIL versus Normal (red), HSIL versus Normal HIV negative (blue), HSIL versus LSIL HIV positive (green) and HSIL versus Normal HIV positive (grey). (**A**) HRHPV positive and negative samples (**B**) HRHPV positive samples only.

The AUROC for the methylation of the gene *CADM1* was 67.2% for HSIL over LSIL and 60.4% for HSIL over normal (Fig. 2A). Also, a comparison of HRHPV positive samples indicated an AUROC of 67.3% for HSIL over LSIL and 61.8% for HSIL over normal (Fig. 2B). Furthermore, similar AUROC (between 60 and 62%) were determined for comparisons between HSIL and normal for HIV negative and positive participants including all samples (Fig. 2A) and HRHPV positive samples only (Fig. 2B). Most significantly, in HIV positive men, *CADM1* methylation distinguished HSIL from LSIL with an AUROC of 73.3% (Fig. 2A) and in HIV positive men with HRHPV infections, the AUC was 78.2% (Fig. 2B).

### Methylation of the gene *MAL* differentiates between HSIL and LSIL for HIV positive men

**Figure 3:**
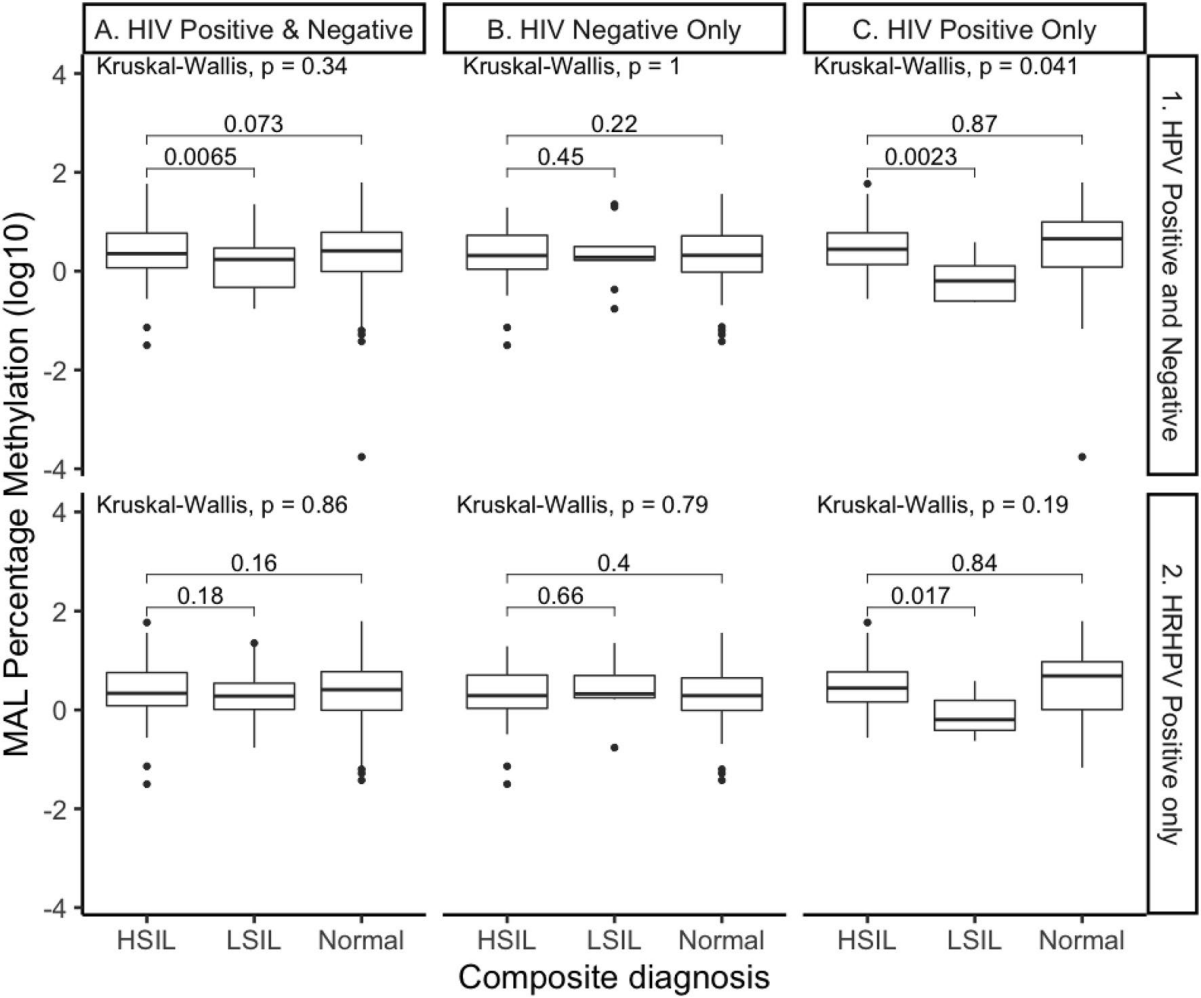
Percentage Methylation of gene *MAL*. All samples represented by HRHPV positivity and HIV status. Comparisons assessed by the nonparametric Wilcoxon test, with error bars corresponding to the first and third quartiles (the 25th and 75th percentiles).

An overall analysis (Kruskal–Wallis) in the percentage methylation of *MAL* identified a significant difference in HIV positive only samples p = 0.041 respectively; Fig. 3, panel C1). Further individual analysis in all samples identified HSIL as significantly different to LSIL (p = 0.0065). This was also observed in HIV positive group (p = 0.0023).

**Figure 4:**
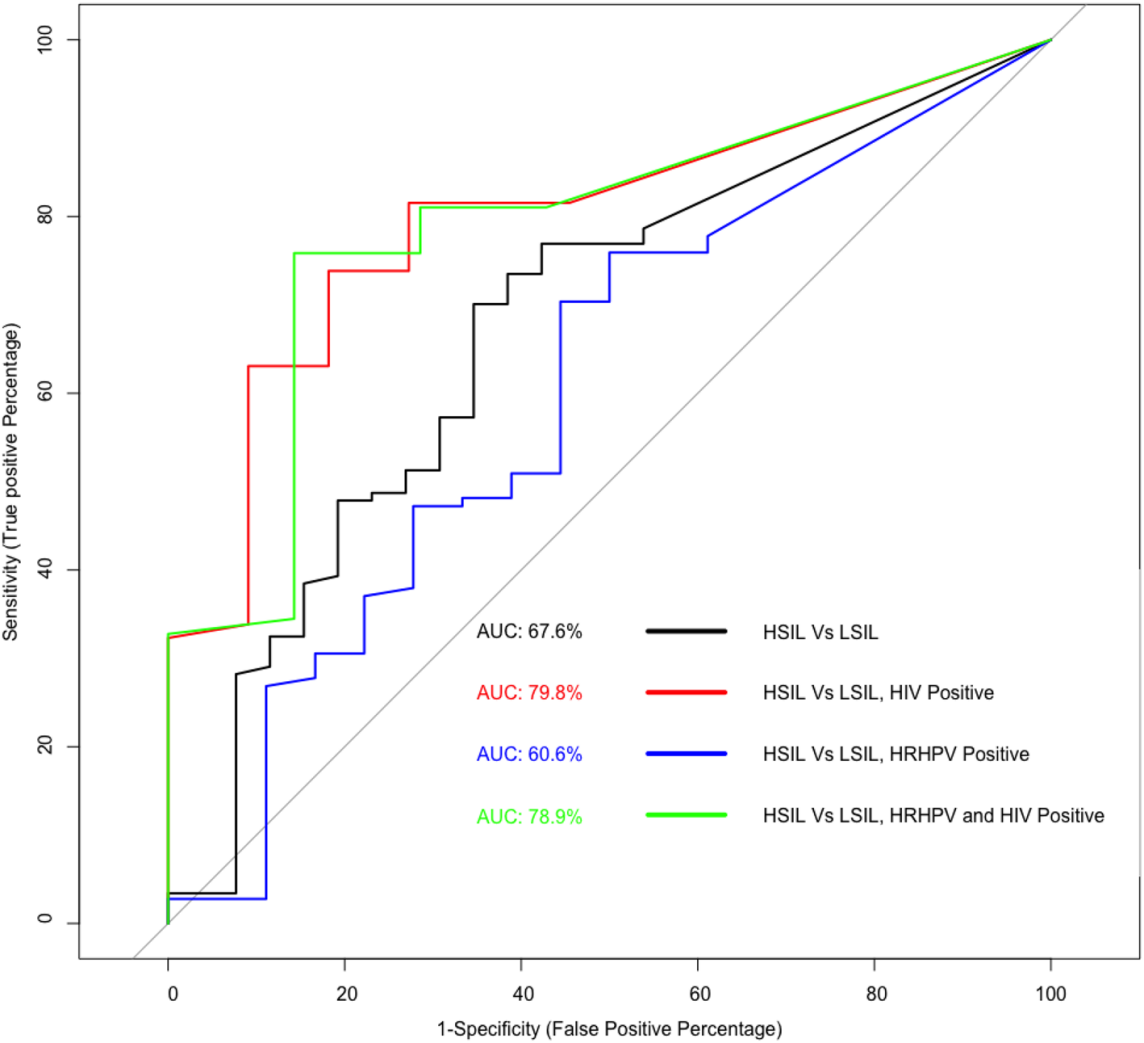
Receiver Operating Characteristic (ROC) curve for the percentage methylation of gene *MAL* for all significant (p, ≤ 0.05) comparison combinations identified in the nonparametric Wilcoxon test. Comparisons include HSIL versus LSIL in all samples (black), HSIL versus LSIL in HIV positive samples (red), HSIL versus LSIL in all HRHPV positive samples (blue) and HSIL versus LSIL in all HIV and HRHPV positive samples (green).

Further comparative analysis of each group (all samples and HRHPV positive samples) stratified by HIV status identified that only HIV positive men had significant differences (p = 0.016) (Fig. 3, panel C1). Within the HIV positive group, methylation in HSIL was significantly higher compared with LSIL in all samples (p = 0.002) (Fig. 3, panel C1) and HRHPV samples (p = 0.017) (Fig. 3, panel C2), however there was no difference when compared with normal samples (Fig. 3, panels C1 and C2); (as observed in the overall group). The AUROC for the methylation of the gene *MAL* was 67.6% for HSIL over LSIL (Fig. 4). Furthermore, HSIL compared with LSIL in HRHPV, and HIV positive anal swab samples indicated an AUROC of 78.9%.

### The methylation of gene *miR124-2* is not predictive of anal HSIL in GBM

**Figure 5:**
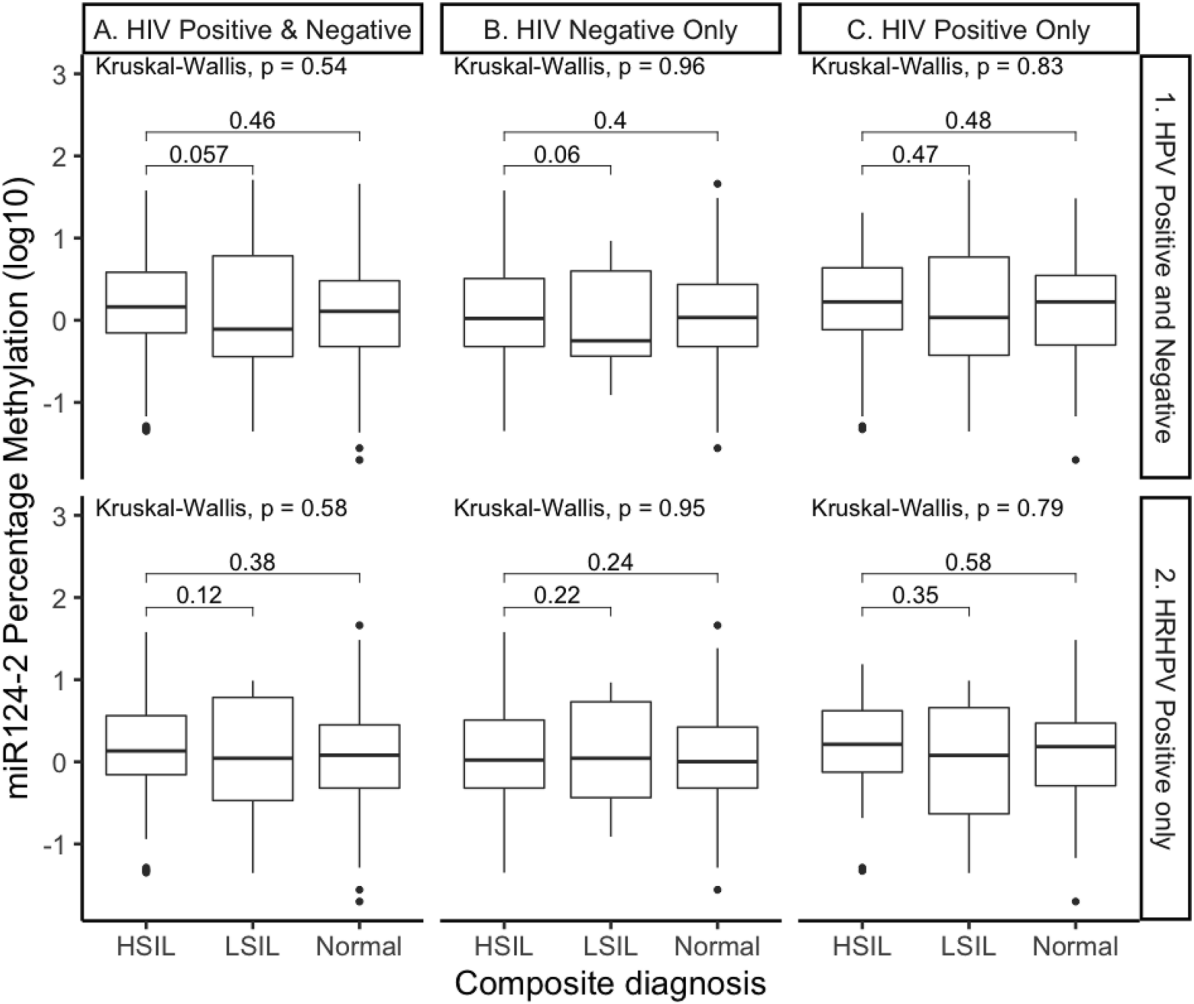
Percentage Methylation of gene *miR124-2*. All samples represented by HRHPV positivity and HIV status. Comparisons assessed by the nonparametric Wilcoxon test, with error bars corresponding to the first and third quartiles (the 25th and 75th percentiles).

The methylation of the promoter region for *miR124-2* compared with composite histologically and cytologically defined anal disease identified no significant differences by anal disease grade between any of the subgroups analysed, including HRHPV positive samples or HIV status (Fig. 5).

### The value of different screening algorithms to predict histologically confirmed HSIL

**Figure 6:**
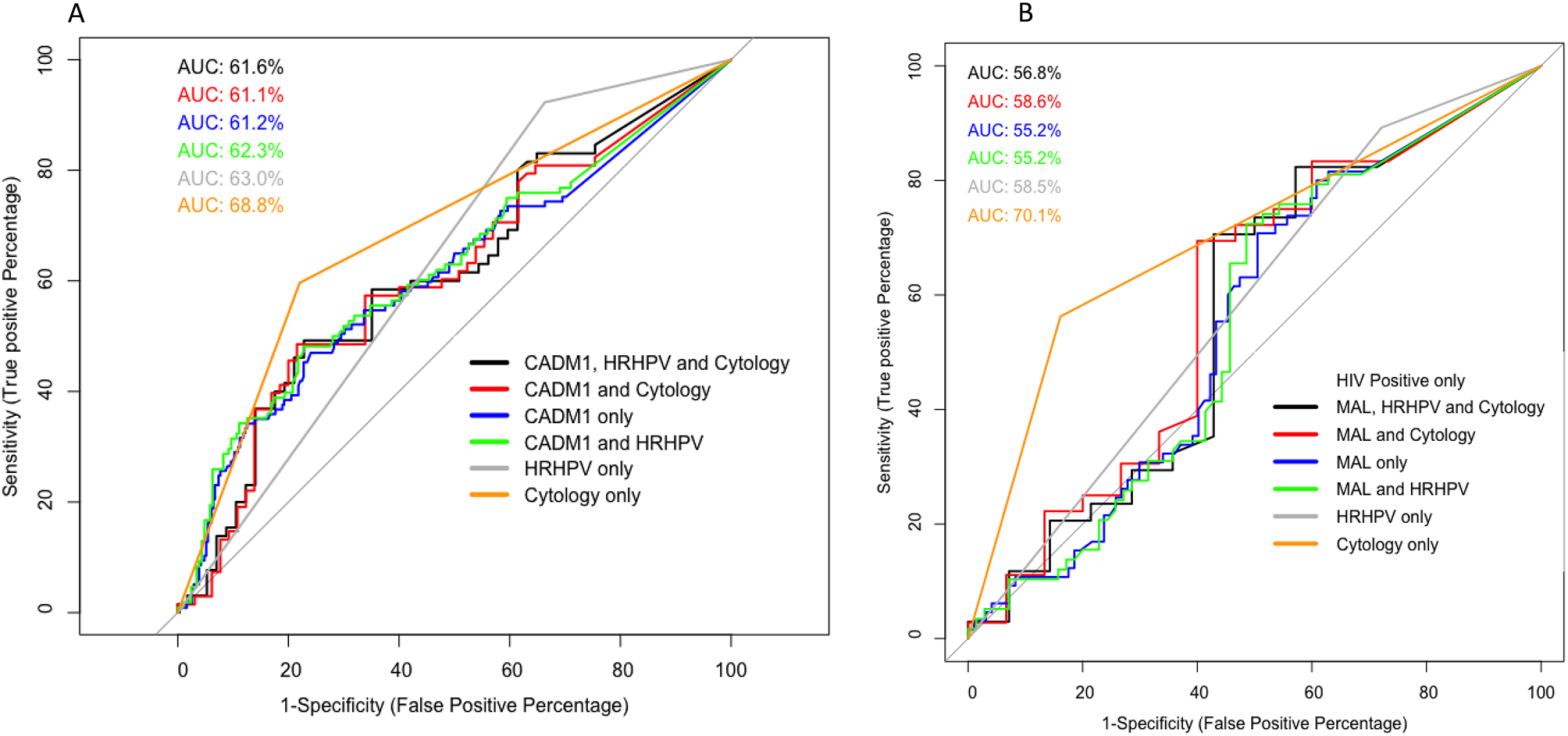
Difference in predictive power for different screening algorithms for anal cancer in men to identify histology defined HSIL from LSIL and normal combined. Algorithms include combinations of gene methylation and/or HRHPV positivity and/or cytology defined HSIL for (**A**) gene *CADM1* in all men and (**B**) gene *MAL* in HIV positive men only. Comparisons for each gene are identical and include gene methylation in men with HRHPV and HSIL cytology (black), gene methylation in men with HSIL cytology (red), gene methylation only (blue), gene methylation in men with HRHPV (green), HRHPV positivity only (grey) and cytology defined HSIL only (orange).

The utilisation of percentage methylation for gene *CADM1* and *MAL* in potential screening algorithms was assessed in combination with and without HRHPV positivity and/or cytological HSIL. Overall, the addition of cytology results had limited effect on the AUROC value of gene methylation (Fig. 6). Cytology alone had the highest AUROC within histologically-confirmed HSIL (68.8%) (Fig. 6A). Using multiple screening markers, *CADM1* in combination with HRHPV positivity had an AUROC value of 62.3% for histologically-confirmed HSIL (Fig. 6A). Within the HIV positive population, the highest AUROC was cytology alone (70.1%) (Fig. 6B). Using a combination of different markers lowered the AUROC value with all markers only achieving 56.8% in histology confirmed HSIL (Fig. 6B). Cut-off values for each methylation marker were also assessed, however no combination tested improved the AUROC percentage above cytology alone (Supplementary Fig. 1).

## Discussion

In the current study, men with HSIL had an increase in the host DNA methylation of CpG sites within promoter regions for the gene *CADM1* and, to a lesser extent, *MAL*. Among HIV positive individuals only, gene *MAL* had significantly less gene methylation in men whose highest grade of disease was LSIL compared with both normal histology and HSIL. This limits the utility of *MAL* as a biomarker of disease detection.

The methylation of CpG sites for both *CADM1* and *MAL* have previously been indicated as potential biomarkers in HPV-related cervical intraepithelial neoplasia (CIN) with reports of 78% specificity and 70% sensitivity in women with HRHPV infections for histological CIN3+^30^. This is the first report of these markers being associated with anal HSIL in men and identifies their potential to be included in combination with other biomarkers for anal cancer screening programs.

The predictive value of *CADM1* methylation for the determination of HSIL indicated that approximately 67% of anal samples would be correctly diagnosed as HSIL with this single marker, with no difference between HIV or HRHPV status.

Other studies of gene methylation markers from anal HSIL and cancer in HIV positive and negative men include *p16, Ki-67, ASCL1, SST, ZIC1, ZNF582* and the HPV E4 gene where significant differences have been reported from tissue samples^31–33^. The methylation of the CpG promoter region of gene *MAL* was observed to be significantly increased only in HIV positive GBM with HSIL (p, ≤ 0.01) as compared with those with LSIL, but not when compared to normal samples or in HIV negative participants. Changes to the expression of the *MAL* gene have previously been identified in several different cancers with early reports identifying *MAL* as a tumour suppressor^34^. Subsequent reports have also identified decreases in MAL expression observed in cervical, colon, breast, stomach, bladder, salivary gland, head and neck and non-small cell lung cancers^35^. In the current report, the significant increase in *MAL* methylation in HSIL compared with LSIL in HIV positive individuals is perplexing, due to the similar methylation patterns between normal and HSIL samples. This unusual progression of changing methylation between normal, low and high grade disease in HIV positive GBM could be due to the MAL protein acting as a raft for other oncogenic proteins, as proposed by Lara–Lemus^35^. However, the limitation of this change to HIV-positive individuals could also be due to HIV-related changes in T-lymphocytes. Individuals infected with HIV experience a sustained hyper immune activation, triggered by changes in T cell dynamics and function^36^. It is possible that during acute infections of HPV, HIV-positive individuals have a varied response to acute HPV infection compared with HIV negative individuals.

Although, the significant findings of *ZNF582* with a 93% specificity for ≥ AIN3 does show promise for the diagnostic potential of methylation markers in the anal mucosa^32^. In addition to these strictly male gender assigned at birth and currently identifying as male studies, further analysis of the gene EPB41L3 and HPV16 in anal samples from HIV positive and negative men and women identified a combined sensitivity of 90.6%, specificity of 50.7% and AUROC of 0.82 in identifying HSIL and cancer from normal tissue samples^37^. It is important to note that these anal specific studies all utilised tissue samples whereas the current study focuses on swab samples. This difference is an important point of difference. If a methylation marker is to be utilised as a screening tool it needs to be sensitive enough to detect disease without the collection of tissue samples, which require highly specialised equipment and personnel. By only focusing on tissue samples the application of the findings are limited until the relevant sample has been assessed. This is where the current study is unique compared to all other anal methylation studies.

However, it should also be noted that determining small changes for low levels of disease is a difficult task which is compounded by utilising swab samples, which contain a high diversity of cellular material, to measure lesion specific changes. Our result indicating higher methylation levels in normal versus LSIL samples could be a consequence of the sample utilised and not differences between disease presentations.

Methylation-based silencing of precursor micro RNAs of microRNA 124 have previously been indicated in various human neoplasms, including the cervix^38,39^, where a significant inverse correlation was observed in the methylation of the promoter region and the presence of *miR124-2* RNA. The same study also demonstrated *in-vitro*, that silencing of *miR124-2* is functionally involved in cervical cancer development^38^. However, in the current study, methylation of the promoter region of *miR124-2* was not found to be different between men with HSIL, LSIL or normal samples. The absence of this important biomarker could be due to the nature of the samples collected, as anal swab samples contain a mixture of normal and diseased cells. Other explanations could be that this is a phenotype specific to the anal canal. Further analyses utilising specific tissue samples from laser capture microdissection techniques and investigations into other precursor micro RNAs for *miR*124 are required to define any roles in the development of HSIL.

Combination algorithms utilising *CADM1,* HRHPV detection and cytology diagnosis marginally improved the predictive value (from 61.2% alone to 61.6% combined) with cytology alone outperforming all other combinations at 68.8% AUROC in differentiating HSIL from LSIL and normal samples. Similar findings were also observed utilising *MAL*, in HIV positive participants. These findings indicate that complex screening algorithms may be required to achieve similar results to those seen in the cervix. For example, if all samples are screened by cytology to filter out all normal samples then CADM1 and HRHPV testing can be added for disease grade diagnosis (between LSIL, HSIL and ASCC) then the predictive value increases to 78.2% (similar to findings in the cervix at 78% specificity and 70% sensitivity in women with HRHPV infections)^30^.

Limitations to this study were the use of single promoter regions for methylation measurements and the absence of mRNA measurements to assess the level of gene silencing at a transcriptional level. Future studies should also assess the mRNA effects due to methylation of all possible promoter regions. The methylation patterns of *CADM1* and *MAL* will be assessed for changes in longitudinal studies of HPV persistence utilising the SPANC cohort of samples. The detection cut off for the internal control was also increased from the published > 32 to > 40 cycle threshold which is a considerably low threshold. However, through modifications to the PCR reactions and confirmation through serial dilutions of the SiHa cell line (inter and intra assays) we confirmed that our modifications increased the assays sensitivity allowing for the increased cycle threshold cut-off.

In conclusion, this study demonstrates methylation of the promoter region for gene *CADM1*, when used in a panel of biomarkers, is a promising candidate for use as part of an anal screening algorithm for HSIL in HRHPV positive GBM. Furthermore, in HIV positive GBM, methylation of the promoter region for gene *MAL* may be important in HIV related immune modulation. It is clear from these findings that biomarkers utilised in the cervix are of limited use within other mucosa tissue sites highlighting the need to further explore new genes of interest specific for HPV related disease of the anal mucosa.

## Methods

### Study population

Anal specimens were obtained as part of the SPANC study. SPANC was a prospective cohort study based in Sydney, Australia, that followed HIV-negative and HIV-positive GBM aged 35 years and older at five visits over a period of three years. Overview of study design, recruitment and protocols have been previously published^5^. Participants were eligible for inclusion if they met the following criteria: had given consent to the use of their samples for further research; had sufficient residual sample remaining after routine SPANC tests were completed; had completed the first three study visits; had baseline samples with satisfactory HPV testing. In this substudy, only baseline samples were utilised.

### Diagnosis and disease classification

Laboratory methods have been previously described^5^. Briefly, cytology was graded as per the Bethesda System criteria^40^ and terms used for cytology reporting. Histology reporting was in accordance with the Lower Anogenital Squamous Terminology (LAST) Project^41^. Final diagnosis for each sample was a composite of cytology and histology reporting (disease results were derived by histology findings from HRA guided biopsy collection. In cases of normal or LSIL cytology grades where biopsies were not collected, or histology unavailable cytology results were utilised). Where multiple samples were collected during HRA, the highest-grade histology findings were used. Composite end points were as follows; All reports of negative were reported as normal, all reports of LSIL was reported as LSIL, all reports of AIN2 and AIN3 were reported as HSIL.

### HPV genotype testing

Baseline residual anal cytology specimens were tested for HPV DNA (including HRHPV types 16, 18, 31, 33, 35, 39, 45, 51, 52, 56, 58, 59, 66 and 68) as previously described^42^, using the Roche Linear Array HPV genotyping test (Roche Molecular Systems, Alameda, CA, United States). As it has been established that Linear Array has a lower sensitivity for several HRHPV genotypes (HPV40, 42, 54 and 68)^43^, a secondary assay was utilised with a higher sensitivity (for these specific genotypes). The Anyplex™ II HPV HR Detection (Seegene, Seoul, South Korea)^44^, a multiplex real-time PCR method that detects 14 HRHPV genotypes (16, 18, 31, 33, 35, 39, 45, 51, 52, 56, 58, 59, 66, 68), was utilised for all specimens. Specimens were considered positive for any HPV type that was detected on either Linear Array or Anyplex™ to achieve a high sensitivity for all HPV genotypes.

### Isolation of nucleic acids for methylation PCR

A 1 mL aliquot taken from a 20 mL cytology sample in Thin Prep PreservCyt medium (Hologic, Inc. Marlborough, MA, USA) was centrifuged at 17000*g* for 15 min, the supernatant removed, and the pellet resuspended in 200 µl of PBS. DNA was extracted on an automated MagNAPure 96 (Roche Diagnostics GmbH, Penzberg, Germany) using the DNA and Viral Nucleic Acid Small Volume Kit (Roche Diagnostics) with a sample volume of 200 µl and an elution volume of 50 µl, according to the manufacturer’s instructions. The concentration of double-stranded DNA was measured with a Qubit® Fluorometer (Life technologies, California, USA).

### Bisulphite DNA modification and quantitative methylation-specific PCR

Extracted DNA underwent bisulphite conversion using the Methylamp™ DNA Modification Kit (Epigentek, New York, USA). Quantitative methylation-specific PCR (qMSP) targeting CpG sites in promoter regions of *CADM1* (promoter region M9), *MAL* (promoter region M1) and *miR124-2* (promoter region 2) were performed using the primer sets and methods previously described^45^, with minor modifications (single-plex PCR reactions, and an increase in magnesium chloride concentration from 3 to 4 mM MgCl_2_). PCR mixtures for the modified protocol contained 2.5 μl of bisulfite modified DNA, primers at 417 nM, probes at 208 nM (labelled with 5’-FAM and 3’-BHQ1), 1 mM of added MgCl_2_, and 1 × Bioline Sensifast Probe Mastermix (containing 3 mM MgCl_2_ per reaction). A segment of the beta actin gene (*ACTB*) was targeted as an internal reference and for quantitation of input DNA^46–48^. A standardised sample of bisulphite-treated DNA extracted from cervical cancer cell line SiHa, (American Type Culture Collection, Manassas, Virginia, USA) containing a single copy of HPV16 per cell, was included as a positive control in each qMSP run. PCR thermocycler conditions were unchanged from the previously published method and were performed on the LC480 LightCycler technology (Roche Diagnostics). The human cell line A549 was utilised as a negative control. SiHa cell line was used as a positive control for variations of each bisulphite modification assay performed and validation of the qPCR methylation analysis for each gene. Briefly, each PCR run had an old bisulphite modified SiHa cell line that had been previously validated for methylation analysis and a new (identical DNA concentration) SiHa cell line that was bisulphite modified at the same time and with the same bisulphite reagents that the samples to be analysed by qPCR that day (in general 9 samples). The objective of this procedure was to analyse the variation in the bisulphite modification procedure between assays and to validate the methylation analysis between different runs (reproducibility of % of methylation of the positive control for each gene). Our results showed very high reproducibility in the % of methylation in the SiHa cell lines for each gene between runs (data not shown).

### Data analysis

A sample was deemed satisfactory if the crossing threshold value for ACTB was < 40. Any sample that was negative for ACTB was not considered for analysis regardless of methylation marker status. The percentage methylation in each individual sample (% meCpG) was calculated as described based on the house keeping gene ACTB.

$$\%\,meCpG = \left( {\frac{1}{{2^{\left( {Cp _{meCpG\,target} - Cp _{ACTB} } \right)}}}} \right) \times 100$$

Percentage methylation for each marker (*CADM1*, *MAL* and *miR124-2*), for each composite disease grade (HSIL, LSIL and normal) was compared using two non-parametric comparative analysis, Wilcoxon test for between groups and Kruskal–Wallis for overall analysis: this was visualised using box and whisker plots. These analyses were further stratified by HRHPV positivity and HIV status.

The diagnostic performance of each methylation marker was evaluated by receiver operating characteristics (ROCs) analyses. Area under the curve (AUROC) was the main measure used to assess the ability of the genes to distinguish/differentiate HSIL from LSIL/normal by using the best cut-off with the maximum sum of sensitivity and specificity. Different screening algorithms were also assessed utilising gene methylation for markers that were significantly associated with HSIL detection, within a sub cohort of HRHPV positive samples and anal cytological HSIL, to predict histologically confirmed HSIL. All %meCpG calculations were performed in Microsoft Excel v16.39 (Microsoft, Redmond, Washington, USA). The results were then analysed and figures produced using the statistical platform R studio (v4.0.1)^49^ and programs ggplots2 (v3.3.2)^50^, ggpubr (v0.4)^51^ and pROC (v1.16.2)^52^ and cutpointr (v1.1.1)^53^ with p values ≤ 0.05 considered significant.

All mentioned methods were performed in accordance with the relevant guidelines and regulations.

### Ethics approval and consent to participate

Ethics approval for the Study of the Prevention of Anal Cancer (SPANC) was granted by the St Vincent’s Hospital (SVH, Sydney, Australia) Human Research Ethics Committee (File Number 09/203).

### Consent for publication

Informed consent was granted by each participant included in the trial through the SPANC recruitment process and detailed in the human research ethics approval (File Number 09/203).

## Supplementary Information

Supplementary Figure 1.

## Data Availability

All data and materials are available within this document and the supplementary files.

## Abbreviations

HPV: Human papillomavirus
LSIL: Low-grade squamous intraepithelial lesion
HSIL: High-grade squamous intraepithelial lesion
ASCC: Anal squamous cell carcinomas
HRHPV: High-risk HPV
GBM: Gay and bisexual men
HRA: High-resolution anoscopy
ROC: Receiver operating characteristic
AUROC: Area under the receiver operating characteristics
SPANC: Study of Prevention of Anal Cancer
LAST: Lower Anogenital Squamous Terminology
qMSP: Quantitative methylation-specific PCR
*CADM1*: Cell adhesion molecule 1
*MAL*: T-lymphocyte maturation associated protein
*miR124*: Micro RNA 124
*ACTB*: Beta actin gene
CIN: Cervical intraepithelial neoplasia
