# Supplementary material for: Gene methylation of CADM1 and MAL identified as a biomarker of high grade anal intraepithelial neoplasia
Source: Sci Rep. 2022 Mar 3;12:3565. doi: 10.1038/s41598-022-07258-5 (PMC8894372; doi:10.1038/s41598-022-07258-5)
Supplement: Supplementary file 1 — Supplementary Figure 1. [file 41598_2022_7258_MOESM1_ESM.docx]

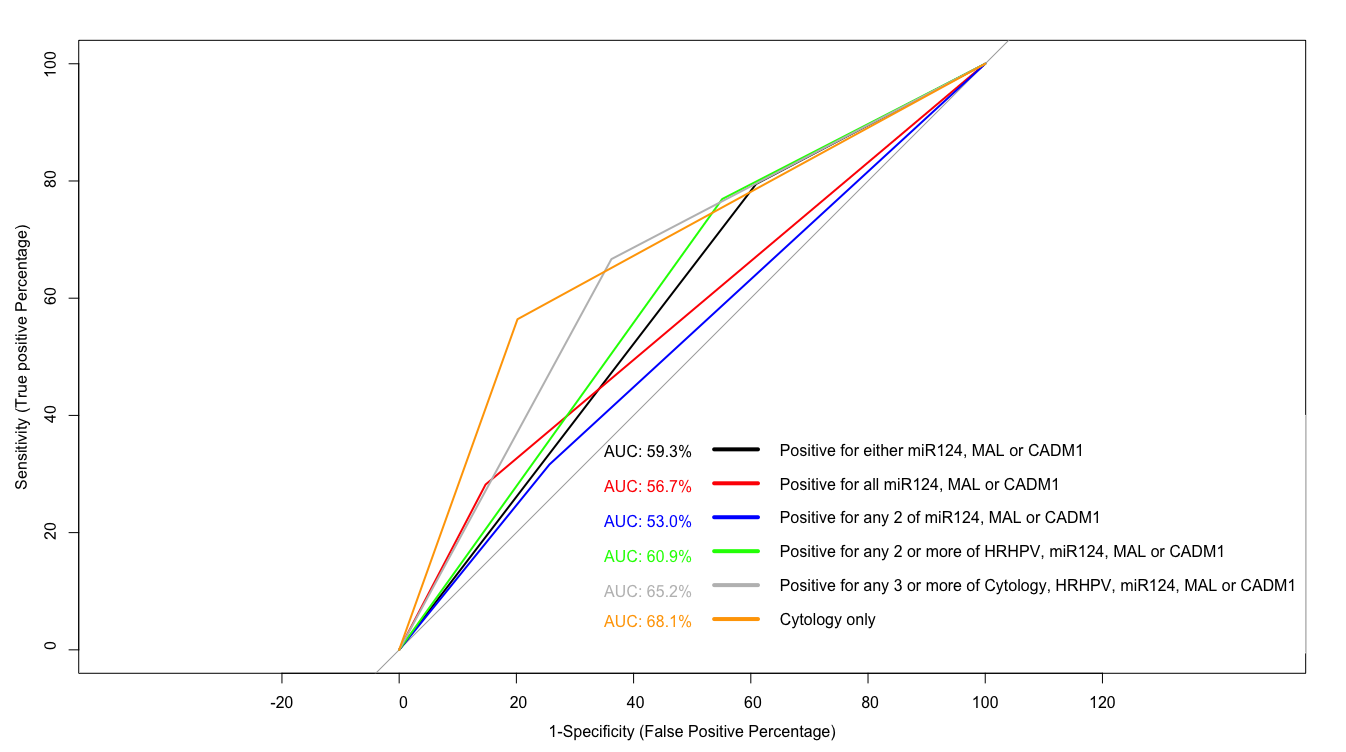


Supplementary figure 1. Assessment of combination testing utilising gene methylation cut-off points. Cut-off points and disease prediction assessments utilised all samples (HIV positive and negative) and considered HSIL (AIN2/3) as positive and LSIL/normal as negative.

(Cytology results also include unsatisfactory results as negative), produced. using R studio (48), cutpointr (52) and pROC (51).
